# MASCAF: A Cable Model Fitting Pipeline for Topologically Complex Surface Meshes

**DOI:** 10.64898/2026.05.10.721501

**Published:** 2026-05-13

**Authors:** Jordan M. R. Fox, Brian J. Fischer, William DeBello, Jose L. Peña

**Affiliations:** 1 Dominick P. Purpura Department of Neuroscience, Albert Einstein College of Medicine; 2 Department of Mathematics, Seattle University; 3 Center for Neuroscience, University of California Davis

## Abstract

We present a free and open-source, semi-automated, topologically robust pipeline for fitting cable models to 3D surface mesh morphology data of neuronal membranes, particularly suited to structures with complex shapes and topological holes. The motivation for this work is the discovery of morphologically complex neural spines on the auditory space-specific neurons of the barn owl (Tyto alba, Tyto furcata), dubbed “toric spines”, notable for their high curvature, branching density, and holes/loops. Multicompartmental simulation software requires morphology to be represented as cable models (e.g., SWC format), yet existing software tools for fitting cable models to complex 3D surface meshes have not produced satisfactory results for toric spines, and loops are generally unsupported. We present the Mesh and Skeleton Cable Fitting (MASCAF) pipeline and software, which fits a cable model (e.g., SWC format) to a surface mesh using mean-curvature flow skeletonization. In this paper, we demonstrate how MASCAF is applied to fit cable models, how loops can be reconstructed in simulations with the Arbor and NEURON simulation software, and how the results can be validated using geometry and simulator-based methods. While non-tree morphologies such as toric spines are neuroanatomically special, our software pipeline provides a cable-model fitting approach for surface mesh data that is topologically robust, deterministic, open-source, and applicable to general morphologies, thereby closing a crucial gap between neuronal imaging and high-resolution simulation.

## 1 Introduction

### 1.1 From Imaging Data to Multicompartmental Simulation

Neuronal morphology plays a fundamental role in shaping electrical signaling, synaptic integration, and neural computation (Rall 1962; Mainen and Sejnowski 1996; London and Häusser 2005). Since the foundational work of Rall and others on cable theory (Rall 1959), it has been well established that dendritic geometry strongly influences input resistance, voltage attenuation, temporal filtering, and nonlinear integration (Agmon-Snir, Carr, and Rinzel 1998; Stuart, Spruston, and Häusser 2016; Koch 1999). Moreover, advances in imaging technologies, particularly serial block-face scanning electron microscopy (SBEM) (Denk and Horstmann 2004) and stimulated emission depletion (STED) microscopy (Hell and Wichmann 1994), now allow reconstruction of neuronal structures at nanometer-scale resolution. While imaging data and mesh representations preserve detailed geometric features, they are not directly compatible with modern multicompartmental simulators such as NEURON (Carnevale and Hines 2006) and Arbor (Cumming et al. 2025), which can provide valuable insights into the relationship between neuronal morphology and function through the *in silico* paradigm. These simulators, using the well-established cable equations (Li, Tetzlaff, et al. 2019; Cazé et al. 2024; Stingl et al. 2025), represent neurons as graphs of isopotential compartments, with morphology typically specified by a cable model composed of connected cylindrical or frustal (frustum-shaped) segments. In such frameworks, realistic morphological specification is important for accurate simulation results.

Fitting cable models, either directly to imaging data or to surface mesh data derived from imaging, is a general challenge (Blumensath and Davies 2011; Liu, Lu, et al. 2016; Peng, Ruan, et al. 2014). A variety of software tools have been developed for these tasks, but naturally, some approaches have limitations that make them inappropriate for particularly complex topologies. In this work, we present a semi-automated, topologically robust pipeline for fitting cable models to 3D surface mesh data, without restricting them to tree-like structures.

The central calculation performed by multicompartmental simulators like NEURON and Arbor is the construction and integration of the cable equations, a system of first-order nonlinear ordinary differential equations that describe changes in membrane potential for the set of isopotential compartments. Equation 1 shows the ODE for a single compartment μ connected to other compartments ν, describing the time evolution of membrane potential $V_{\mu }$. Specific membrane capacitance, denoted $c_{m}$, has units of capacitance per area. The term $i_{\mu }$ has units of electrical current per area and accounts for membrane mechanisms specific to compartment μ, such as passive currents and voltagegated ion channels. External current injection into compartment μ is denoted $I_{\mu }^{\text{ext}}$, and $A_{\mu }$ is its surface area. The sum in the last term accounts for current contributions from connected compartments ν, with $g_{\mu ,\nu }$ denoting the specific conductivity of the connection in units of conductance per area, which depends on the axial resistivity and the geometry of compartments μ and ν.


(1) $$c_{m}\frac{dV_{\mu }}{dt}=i_{\mu }+\frac{I_{\mu }^{\text{ext}}}{A_{\mu }}+\sum_{\nu }g_{\mu ,\nu }\left(V_{\nu }-V_{\mu }\right)$$

The geometry and connectivity of the cable graph strongly influence the cable equations and, thus, the membrane potential produced by the simulator. The goal of MASCAF is to provide more realistic cable models for complex morphologies and, therefore, increase the quality of multicompartmental simulation results and predictive capabilities.

The MASCAF pipeline assumes 3D surface mesh data as input (edges and triangular faces defined on a 3D node basis, e.g., the Wavefront OBJ format (Wavefront Technologies 1990)). For morphologies derived from imaging data, this may require an intermediate membrane-tracing step, typically tracing the neuronal membrane on each slice and converting the result to a triangle mesh. As such, combining MASCAF with a membrane-tracing process to produce surface meshes constitutes a complete pipeline from imaging data to multicompartmental simulation. Many software tools exist for semi- or fully automated tracing of cell features in imaging data (Acciai, Soda, and Iannello 2016).

Cable models typically encode neuronal morphology as an *arborescence*: in graph theory, a directed, rooted, acyclic tree. The SWC data format (Mehta et al. 2023) is the most widely used general-purpose cable model format. An SWC model describes an arborescence as a list of vertices (also often called *nodes* or *samples*), each with the following parameters:

- unique index (integer)
- tag (integer).
- spatial coordinates x, y, z (float)
- local radius (float)
- parent index (integer)

**Figure 1:**
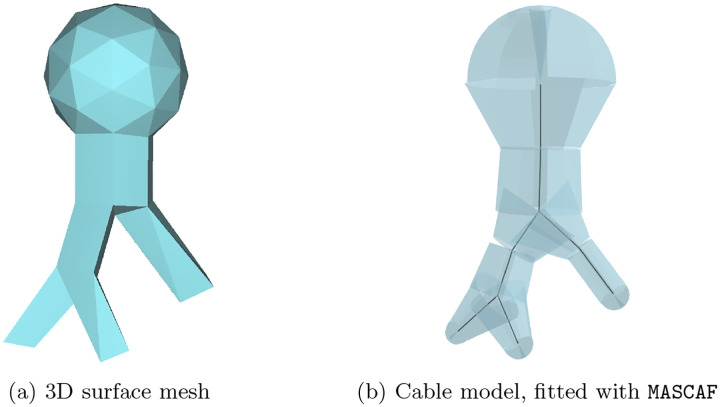
A simple example of a 3D surface mesh and its cable model.

In a valid SWC model, every non-root node has exactly one parent, implicitly enforcing acyclicity. A simple example of a mesh and corresponding cable model is shown in Figure 1. MASCAF produces cable models in SWC format with cycle metadata appended to the file header, which can be read when constructing the simulation.

### 1.2 Motivation: Toric Spines in the Barn Owl Auditory Pathway

The development of the MASCAF pipeline is motivated by the discovery of uniquely shaped dendritic spines, termed toric spines, on auditory space-specific neurons (SSNs) of the barn owl (*Tyto alba*, *Tyto furcata*) (Sanculi et al. 2020). Auditory receptive fields in the midbrain emerge through integration of sound frequency and interaural time and level differences (ITDs, ILDs) (Peña and Konishi 2001; Peña and Konishi 2002; Fischer, Anderson, and Peña 2009). SSNs play a central role, coding for sound source location in the visual field (Spezio and Takahashi 2003; Keller and Takahashi 2005; Singheiser, Gutfreund, and Wagner 2012). Found in the owl’s inferior colliculus (IC) external nucleus (ICx), these neurons collectively comprise a topographic map of auditory space (Eric I. Knudsen and Konishi 1978): stereotaxic coordinates within the brain map continuously onto azimuth and elevation in the visual field. The ICx is also a major site of experience-dependent plasticity, demonstrating adaptive remodeling and recalibration of auditory space representations during development and sensory learning (Gold and Eric I. Knudsen 2000; DeBello, Feldman, and Eric I. Knudsen 2001). As such, the barn owl has served as a foundational animal model for studies of audition, sound localization, neuroethology, and experience-dependent plasticity for more than five decades (Payne 1971; Konishi 1973; Eric I Knudsen and Konishi 1979; Moiseff and Konishi 1981).

Toric spines, as their name suggests, commonly exhibit topological loops, though not always. They have other distinct morphological features compared to typical dendritic spines, including overall larger size, wide necks, a lack of distinct spine heads, and irregular thickness. Importantly, while a typical dendritic spine has only one synapse, a toric spine can have numerous synapses (up to 49) derived from multiple axons (up to 11). Imaging of juvenile barn owl brains shows developing toric spines with consistent features; complete loops are formed during development and are found in adults. It is known that the map of auditory space in the ICx is learned and will adapt to a persistent systematic offset of the visual field (Eric I Knudsen and P. F. Knudsen 1989), but little is known about the function of toric spines specifically, much less their loops. For further details on toric spines, refer to the original publication (Sanculi et al. 2020). A rigorous examination of the relationship between toric spine morphology and synaptic integration is part of our future work.

**Figure 2:**
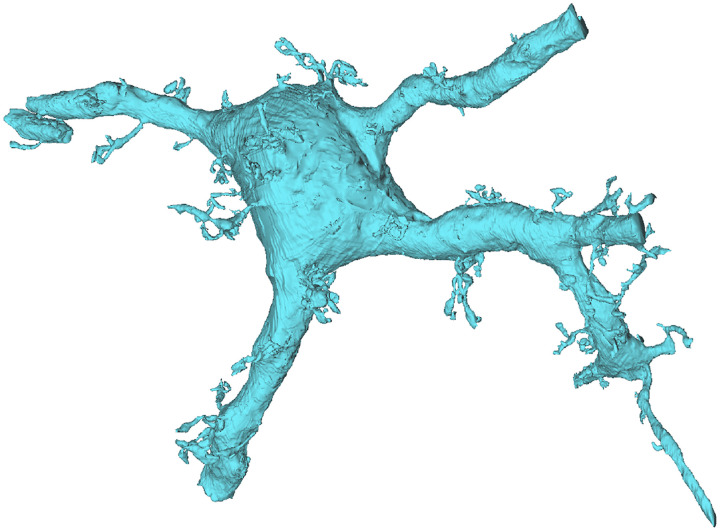
Mesh surface reconstruction of auditory space-specific neuron in the barn owl midbrain, compiled from electron microscopy data. Large dendritic processes extend beyond the captured field and appear cut off. Toric spines are visible as thorny structures covering the surfaces of the dendrites and soma.

**Figure 3:**
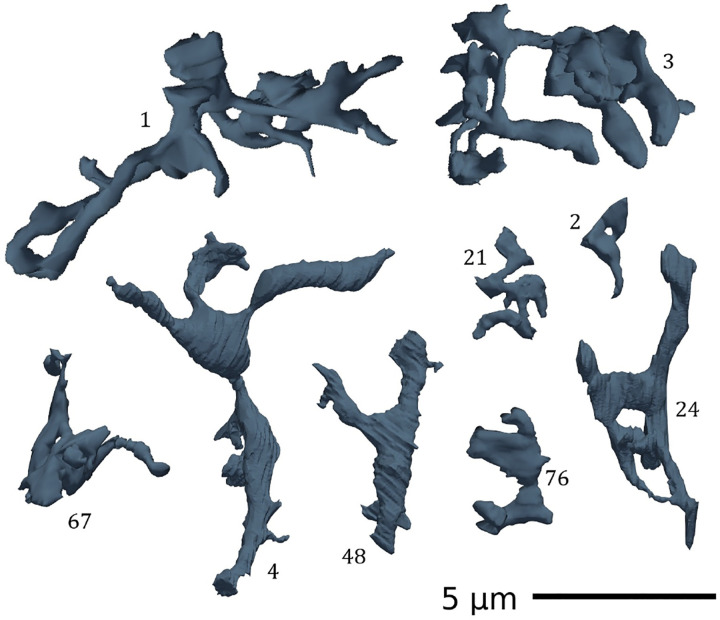
Surface mesh reconstructions of nine selected toric spines from the barn owl ICx neuron shown in Figure 2. Each spine exhibits complex morphology including multiple necks, high curvature, and topological holes (loops).

On a single barn owl ICx SSN chosen for high-resolution surface reconstruction from SBEM data, shown in Figure 2, 72 spines were identified, all classified as toric. The toric spines on this cell were observed to have anywhere from zero to nine holes, as well as from one to three necks per spine. Of the 72 toric spines observed, 9 were selected for detailed reconstruction and simulation. Their 3D surface meshes are shown in Figure 3.

Software tools for neuron modeling often sensibly assume an arborescent structure, but the loops of toric spines violate that assumption. The simulators NEURON and Arbor conveniently allow the establishment of direct self-connections via gap junction-like mechanism, and, therefore, the core modeling challenge is in fitting a valid cable model to the surface mesh. The methods we present for skeletonization and cable model fitting were developed for toric spines, which differ from common whole-neuron morphologies in overall scale and geometry, but MASCAF can be applied similarly to any valid 3D surface mesh, as demonstrated in Section 3.

Conceiving of a pipeline to prepare toric spine morphologies for multicompartmental simulation, we identified the following desired characteristics.

- The pipeline must be capable of topologically robust skeletonization (1D midline calculation) of 3D surface mesh data. It must be able to accurately skeletonize complex, high-curvature, and cyclic geometries. The number of cycles in the cable model must equal the topological genus of the surface mesh.
- The pipeline must output a cable model in SWC format or an equivalent format, from which an accurate morphological representation can be built in the simulator.
- The pipeline’s skeletonization phase should ideally consist of direct geometric operations, rather than relying on global optimization or deep learning approaches.
- The pipeline should take only the mesh as input, without requiring additional geometric data.
- The pipeline ought to consist entirely of free and open source software.

Despite the focus on anatomical accuracy via geometric/topological fitting, it is crucial to note that the utility of the cable model lies in its electrical representation of the biological structure, not a geometric one. A cable model provides the multicompartmental simulator software with a morphological basis for modeling the structure’s electrical properties. As such, we prioritize the accuracy of the electrodynamical simulations over geometry and visuals.

### 1.3 Contributions

The main contribution of this paper is the Mesh and Skeleton Cable Fitting (MASCAF) pipeline (Fox 2026), which fits a cable model to a 3D surface mesh.

1. We explain how the CGAL-TSMS algorithm is implemented to provide a robust, computationally efficient approach for skeletonizing complex meshes, including those with cycles. A high-level description of the process is given in Section 2.2.
2. We define a flexible computational pipeline for fitting a cable model to the combined mesh and skeleton data.
3. We validate the resulting cable models using geometry- and simulation-based measures.

By enabling the conversion of high-resolution mesh reconstructions into multicompartmental simulation models without reliance on proprietary software, this pipeline lowers technical and financial barriers to morphology-driven computational neuroscience.

## 2 Methods

This section describes steps of the MASCAF pipeline for fitting a cable model to a 3D surface mesh. A flowchart of the MASCAF pipeline is shown in Appendix A.

### 2.1 Input Data and Mesh Pre-processing

Mesh data is eligible for the CGAL-TSMS algorithm if and only if it is a single component, has no self-intersections, and no boundaries (i.e., is “watertight”). If any of these prerequisites are not met, the operation will return an error before running.

The MASCAF Python code offers two mesh preprocessing operations using CGAL: repair (Ohori 2026), and simplification (Cacciola et al. 2026). If the original mesh has a higher resolution than is needed for skeletonization, one may apply the simplification operation to reduce the number of vertices and edges. This will speed up the subsequent skeletonization operation, which scales with the number of mesh vertices. In our case, some meshes converted directly from contour data had several hundred thousand edges, and simplifying them to a few thousand edges greatly sped up operations without sacrificing fidelity.

### 2.2 Mean Curvature Flow Skeletonization via CGAL-TSMS

The central algorithm in CGAL-TSMS is mean-curvature flow skeletonization (MCF/MCFS), which iteratively displaces vertices according to local curvature until the mesh collapses to a single curve. CGAL-TSMS can be applied within the MASCAF Python code, in the CGALLab program (GUI), or programmatically in C++ code using the CGAL library. A thorough description and analysis of MCFS can be found in the original paper (Tagliasacchi et al. 2012). A brief and application-focused description of the MCFS process is as follows.

Consider a map $p:U\subset R^{2}\to R^{3}$ where p(u,v)=(x(u,v),y(u,v),z(u,v)) and U is a closed domain in the u,v plane. We denote our closed surface S as S≡{p(u,v)∣u,v∈U}, which encloses a volume Ω, so S=∂Ω.

*Mean curvature flow* (MCF) is a dynamical process applied to the surface S wherein the time rate of change of the surface S is equal to the local antinormal vector −n (pointing into Ω) scaled by the local mean curvature $H=\left(k_{1}+k_{2}\right)/2$, where $k_{1}$ and $k_{2}$ are the maximum and minimum local curvatures (real numbers).


(2) $$\dot{S}=-Hn=-\Delta_{S}p$$

In practice, however, the vector Hn is challenging to compute. Instead, MCFS invokes the Laplace-Beltrami operator, $\Delta_{S}$, a generalization of the standard Laplacian operator to curved subspaces.


(3) $$Hn=\Delta_{S}p\approx Lp$$

Here, L is the discrete approximation to $\Delta_{S}$ used in the calculation. The skeleton solution $p^{*}$ is interpreted as having infinitesimal volume and surface area, and so mean curvature flow halts at that point.


(4) $$Lp^{*}=0$$

A surface mesh $M_{S}$ has vertices $V=\left[v_{1},v_{2},\ldots ,v_{n}\right]$, the locations of which constitute a discrete approximation of the function p. The CGAL-TSMS algorithm updates vertex positions with an implicit Euler step, $V^{t}\to V^{t+1}$ until total change in V is below a convergence threshold. Further details about the solution process are discussed in the original publication (Tagliasacchi et al. 2012).

To understand the parameterization of MCFS relevant to MASCAF, we can consider the total “energy” of MCF for a configuration V at time t,

(5) $$E={\left\|w_{L}LV^{t+1}\right\|}^{2}+w_{H}\sum_{i}{\left\|v_{i}^{t+1}-v_{i}^{t}\right\|}^{2}+w_{M}\sum_{i}{\left\|v_{i}^{t+1}-\mu \left(v_{i}^{t}\right)\right\|}^{2},$$

which is consequently minimized during MCF. Here, $w_{L},w_{H}$, and $w_{M}$ are weight parameters, and μ(⋅) is a map from a vertex to its nearest Voronoi pole. Voronoi poles constitute a sparse (and often noisy) approximation of the mesh’s midline in the form of a point cloud; they are useful here as soft constraints, guiding V toward the midline, but do not constitute a solution to MCFS on their own. The three weights are constrained by a partition-of-unity property; only $w_{H}$ and $w_{M}$ must be set by the user, and $w_{L}$ is computed internally. Intuitively, a larger weight value emphasizes the corresponding energy term’s contribution to the solution of MCFS: a larger $w_{H}$ leads to smaller changes per time step, and a larger $w_{M}$ produces vertices closer to Voronoi poles.

The CGAL-TSMS C++ implementation has the following exposed parameters.

- is medially centered (boolean): Is $w_{M}>0$ ?
- quality_speed_tradeoff (float): $w_{H}$
- medially_centered_speed_tradeoff (float): $w_{M}$

In MASCAF, is_medially_centered should generally be set to True because increasing $w_{M}$ provides a solution with vertices nearer to the mesh’s midline. The values of $w_{H}$ and $w_{M}$ affect skeleton quality (smoothness and branching density), and we cannot provide a single parameterization that guarantees high-quality skeletons in general. For all our toric spine meshes, skeletons were generated with $w_{H}=0.5$ and $w_{M}=5$.

#### 2.2.1 Skeleton Data: Polylines and the Skeleton Graph

The skeleton Σ computed by CGAL-TSMS is specified in a format dubbed *polylines*: a set of m ordered lists of 3D points:

(6) $$\Sigma ={\left\{\left(x_{1}^{i},y_{1}^{i},z_{1}^{i},x_{2}^{i},y_{2}^{i},z_{2}^{i},\ldots ,x_{N_{i}}^{i},y_{N_{i}}^{i},z_{N_{i}}^{i}\right)\right\}}_{i=1}^{m}\;={\left\{\left(x_{1}^{i},x_{2}^{i},\ldots ,x_{N_{i}}^{i}\right)\right\}}_{i=1}^{m}$$

where $x_{j}^{i}=\left(x_{j}^{i},y_{j}^{i},z_{j}^{i}\right)$ is a single 3D point (the jth point in polyline i).

The locations of branch points (degree 3 or more) and terminal points (degree 1) can be deduced from polylines data, which allows us to form a useful object, the *skeleton graph*. The first and last coordinates in each polyline, $x_{1}^{i}$ and $x_{N_{i}}^{i}$, are individually either a branch point or a terminal point. If the coordinate of one of these points is equal to that of another point in a different polyline, then the two polylines are considered connected at that location, and so it marks a branch point; if not, then it is a terminal point. Points with $1<j<N_{i}$ are intermediate, degree 2, and no degree 0 points ought to exist. This is sufficient information to construct the skeleton graph, which is an undirected single-component graph $G_{\Sigma }$ with vertices Σ, and edges describing the connectivity structure implicit in the ordering of Σ.

#### 2.2.2 Skeleton Quality

Evaluating the quality of a 1-dimensional curve skeleton for a 3-dimensional volume is not straightforward (Blum 1967). The *medial axis* of an N-dimensional volume can be defined as the set of centers of maximal inscribed balls, which is generally a (N−1)-dimensional manifold (Siddiqi and Pizer 2008). In other words, the canonical medial axis transform of a 3D volume produces a 2D manifold, not a 1D curve. Because of this, there is no optimal cable model reconstruction for a 3D volume in general. We further discuss this issue in Section 4.1.

A high-quality result of CGAL-TSMS is a skeleton that A) is near to the mesh’s midline throughout the whole volume, and B) has optimal branching density.

- Despite setting $w_{M}>0$, the skeleton produced by CGAL-TSMS will likely not have all points centered on the midline. Radius fitting is generally robust to slight deviations, but if a cable model vertex is placed entirely outside the mesh surface, then radius fitting can fail.
- Errors from sub-optimal branching density (i.e., spurious branches over-filling the mesh volume) can only be evaluated after radii are fitted, as it entails measuring the volumetric overlap of the union of cable model segments with the original volume. Since MASCAF computes local radii after the skeletonization stage, one may need to estimate optimal branching density from the skeleton by comparing to the mesh surface. MASCAF includes a graph-pruning option, which can be helpful to delete some spurious branches.

In either of these cases the user may adjust the parameters $w_{H}$ and $w_{M}$, and rerun CGAL-TSMS to produce a new skeleton. However, it can sometimes be challenging to find a parameterization of CGAL-TSMS that provides a skeleton near the midline without creating spurious branching, since increasing the parameter $w_{H}$ generally improves skeleton accuracy, but also increases branching density. In the next section, we introduce an optimization scheme that refines the positions of morphology graph vertices after the initial basis is constructed from the skeleton.

### 2.3 Cable Model Fitting

Construction of the cable model consists of two steps: constructing the base morphology graph $G_{M}$ from the skeleton graph $G_{\Sigma }$, and fitting local radii by measuring local distances to the mesh surface. A pseudocode description of the algorithm is included in Appendix C.2.

#### 2.3.1 Basis Construction & Optimization

The morphology graph $G_{M}\left(V_{M},E_{M}\right)$ is a non-directed graph that will form the spatial and structural basis in the final cable model. The vertices $V_{M}$ are instantiated with position values. Typical skeleton data from CGAL-TSMS has a high spatial density of points, much higher density than is useful for the ultimate cable model, so we resample $G_{\Sigma }$ to compute the initial $G_{M}$. We first identify a set of all terminal and branch vertices as C={v∈V∣deg(v)≠2}, then tabulate the set of N unique non-branching sections, ${\left\{g_{\Sigma }^{k}\right\}}_{k=1}^{N}$. A non-branching section is a maximal edge-connected subgraph of one of two types: (*i*) a path whose endpoints lie in C and whose interior vertices all have degree 2, or (*ii*) a cycle whose vertices all have degree 2. The skeleton graph $G_{\Sigma }$ is decomposed into non-branching sections $g_{\Sigma }^{k}$ by tracing all maximal paths emanating from critical vertices and then tracing any remaining cycles. This yields an edge-disjoint decomposition of the graph into maximal non-branching sections: $G_{\Sigma }=\overset{N}{\underset{k=1}{⋃}}g_{\Sigma }^{k}$. Given a global maximum edge length parameter ℓ, each $g_{\Sigma }^{k}$ with arc-length $d\left(g_{\Sigma }^{k}\right)$ is resampled to have $\left\lceil d\left(g_{\Sigma }^{k}\right)/\ell \right\rceil +1$ evenly spaced vertices, including the endpoints, to give a new non-branching section $g_{M}^{k}$. Finally, the morphology graph is the union of all resampled sections, $G_{M}=\overset{N}{\underset{k=1}{⋃}}g_{M}^{k}$. This gives a robust process to compute basis nodes from the skeleton while maintaining global control over edge length via ℓ.

Basis optimization, which refines the positions of $V_{M}$, proceeds in two phases: vertex snapping and vertex forcing. By default, branch and terminal nodes are not moved by these phases. Vertex snapping moves any skeleton vertices located outside of the mesh to the nearest point inside the mesh surface. A vertex $v_{i}$ is moved from outside $M_{S}$ to a new location

(7) $$v_{i}^{\prime }=v_{i}+(1+\epsilon )\left(\sigma_{S}\left(v_{i}\right)-v_{i}\right)$$

inside $M_{S}$, where $\sigma_{S}(\cdot )$ is a map from $v_{i}$ to the point on S nearest to $v_{i}$, and ϵ is a small extension factor ≤ 0.1 to ensure $v_{i}^{\prime }$ is inside the mesh volume. The function $\sigma_{S}(\cdot )$ is computed using trimesh (Dawson-Haggerty et al. 2019). This approach is computationally inexpensive, especially since the number of vertices outside the mesh surface is typically small, and may be sufficient to proceed with radius fitting. However, while all points are now inside the surface, the skeleton still likely does not represent the mesh’s true midline. If even greater quality is necessary, we continue to the second optimization phase.

Vertex forcing uses an iterative method to move any vertices that are already inside the mesh closer to the true midline. This process distributes $N_{R}$ rays evenly on a unit sphere centered at the vertex $v_{i}^{t}$, denoted as unit vectors ${\overset{ˆ}{x}}_{j}$. For each ray, the scalar distance $d_{j}$ to the nearest intersection with the mesh surface is computed; a vertex near the midline will have roughly equal $d_{j}$ in all directions, while an off-center vertex will be pulled toward the more distant surfaces. The resulting centering direction is the normalized sum of the outward ray directions weighted by the reciprocal of their surface distances,

(8) $${\overset{ˆ}{F}}_{i}^{t}=\frac{\sum_{j=1}^{N_{R}}{\overset{ˆ}{x}}_{j}/d_{j}}{\left\|\sum_{j=1}^{N_{R}}{\overset{ˆ}{x}}_{j}/d_{j}\right\|},$$

which points from $v_{i}^{t}$ toward the midline. To suppress jitter and maintain spatial coherence, the centering direction is blended with a smoothing direction ${\overset{ˆ}{S}}_{i}^{t}$, defined as the unit vector from $v_{i}^{t}$ toward the centroid of its neighbors in $G_{M}$:

(9) $${\overset{ˆ}{S}}_{i}^{t}=\frac{{\bar{v}}_{i}^{t}-v_{i}^{t}}{\left\|{\bar{v}}_{i}^{t}-v_{i}^{t}\right\|},\;{\bar{v}}_{i}^{t}=\frac{1}{\left|𝒩(i)\right|}\sum_{k\in 𝒩(i)}v_{k}^{t},$$

where 𝒩(i) denotes the set of neighbors of $v_{i}^{t}$ in $G_{M}$. At iteration t, a vertex $v_{i}^{t}$ is moved to position

(10) $$v_{i}^{t+1}=v_{i}^{t}+h\left[(1-\alpha ){\overset{ˆ}{F}}_{i}^{t}+\alpha {\overset{ˆ}{S}}_{i}^{t}\right]$$

where h is a step size, α∈[0,1] is a smoothing weight, and $N_{R}$ is the number of rays, all set by the user. This calculation scales like $O\left(N_{R}|\Sigma |\right)$; we find that relatively small iteration counts (< 10) produced satisfactory refinement.

#### 2.3.2 Computing Radii

The local radius of each vertex in $V_{M}$ is computed via the following process. Consider a target vertex $v_{i}\in V_{M}$. For each adjacent edge $e_{ij}$, we define a cross-section plane using the edge 3D vector $e_{ij}$ as the defining normal, perpendicular to the plane. Where the plane intersects the mesh, a polygonal cross-section with area A is computed using standard mesh manipulation algorithms (trimesh (Dawson-Haggerty et al. 2019)). Different radius calculation strategies were evaluated, and the most accurate and robust was to use the radius of a disk with same area as the cross-sectional polygon, therefore $r(A)=\sqrt{A/\pi }$. This process is repeated for each edge $e_{ij}$ connected to $v_{i}$, and the assigned radius $r_{i}$ is a reduction of those, $f\left(\left\{r_{j}\right\}\right)$, typically mean (the default) or median. This averaging allows for more robust radius assignment, since $r=\sqrt{A/\pi }$ can be noisy for complex morphologies.

### 2.4 Segment Overlap and Surface Area Normalization

A fundamental problem in geometric fitting of cable models arises from volumetric and surface-area overlap at vertices with more than two edges (branch nodes). At every vertex in a cable model with more than two edges, there is a systematic positive overlap in volume and surface area of the connected segments. Depending on attributes of the morphology like branching density, curvature, and compartment shape, the errors introduced by these overlaps may or may not require attention. Indeed, for many whole-neuron morphologies with thin neurite processes much longer than their radius, the contributions of overlaps to volume and surface area relative to the total are often negligibly small. However, if the mesh has wide compartments, high curvature, and high branching density, the overlaps can contribute substantially. In toric spines, the volume and surface area of the cable model (via geometric union) are consistently greater than those of the mesh, occasionally up to 2- or 3-fold larger. We established with Equation 1 that compartment surface area has a significant effect on electrical simulations, augmenting or suppressing the effects of external currents. As such, MASCAF includes a final normalization step to treat overlap effects.

We introduce a global scaling parameter that multiplies all radii of the cable model, and optimize it so that the model’s total surface area, including overlapping areas, matches that of the mesh. As such, relative scales between radii within the same model are preserved, meaning, for instance, ratios of membrane conductances between compartments are preserved.

### 2.5 Simulator Integration

Since the SWC format requires an arborescence, a rooted acyclic tree graph, cycles in the morphology graph must be resolved prior to export. First, all cycles in the morphology graph $G_{M}$ are detected using standard graph traversal algorithms (Hagberg, Schult, and Swart 2008). On each detected cycle $g_{\circ }^{k}\subset G_{M}$, one non-branching vertex v is selected. This vertex is duplicated to create a clone $v^{\prime }$ with identical spatial coordinates and radius. One of the edges connected to v is then updated to terminate at v instead of $v^{\prime }$. The two duplicate vertices are distinct in the cable model and are not connected by an edge. This operation breaks the cycle while preserving geometric embedding. Metadata describing the broken cycle is stored in the SWC file header, which provides the indices of the vertex at which the cycle was broken, and its clone. Or, one can simply check if any two vertices share a coordinate, and unite them if so.

The original loop $g_{\circ }^{k}$ can be restored in the simulator by adding a direct connection between v and $v^{\prime }$. To do this in Arbor, we use the existing gap junction functionality, which connects two points on the model with a conductance parameter. A similar procedure can be done in NEURON. We set the conductance of this connection (in siemens) to the reciprocal of axial resistance to model full cytoplasmic continuity.

## 3 Results

**Figure 6:**
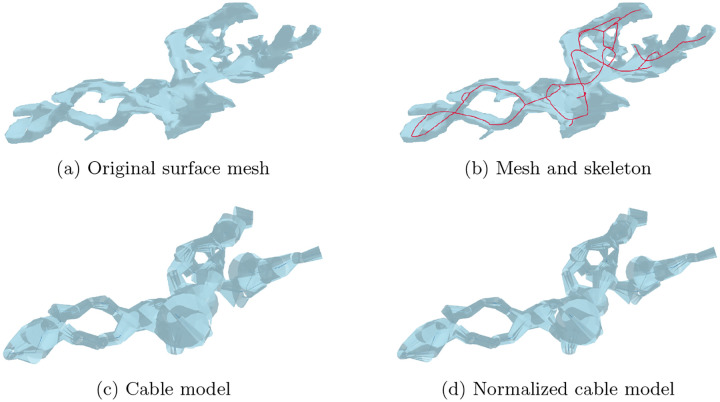
Toric spine #1. (a) Original surface mesh. (b) Mesh with CGAL-TSMS skeleton overlaid. (c) Fitted cable model. (d) Normalized cable model after surface area scaling.

Selected results of MASCAF applied to toric spines are shown in Figure 6 and in Appendix D.

**Figure 7:**
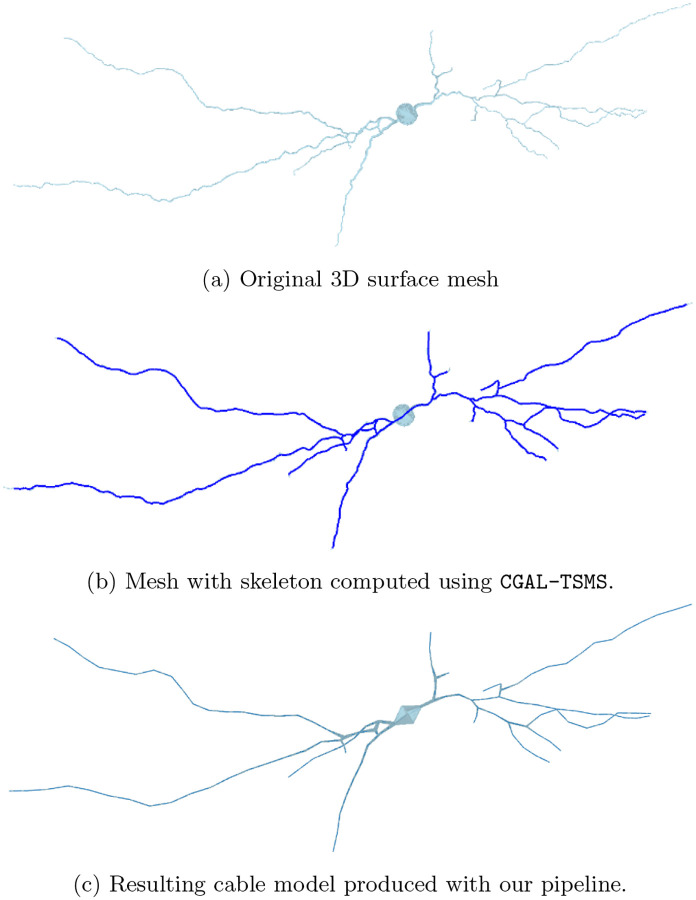
Three steps illustrating MASCAF applied to the surface mesh of a human cortical interneuron (middle temporal gyrus).

To illustrate the applicability of MASCAF to full neuronal morphology data, we include results for a human neuron, shown in Figure 7. The morphology mesh data for this human cortical interneuron (middle temporal gyrus), reconstructed by the Allen Institute, was provided by NeuroMorpho.org (Koch and Jones 2016; Tecuatl, Ljungquist, and Giorgio A Ascoli 2024; Giorgio A. Ascoli, Donohue, and Halavi 2007).

### 3.1 Geometric Validation

To evaluate geometric accuracy of results, we compute a surface mesh from the morphology graph $G_{M}$ and compare that to the original mesh $M_{S}$. Consider the morphology graph $G_{M}\left(V_{M},E_{M}\right)$. For each pair of vertices $v_{i},v_{j}\in V_{M}$ that share an edge $e_{ij}$ with length $\left|e_{ij}\right|$, we construct a *segment* geometry, a frustum with radii $r_{i},r_{j}$, which has volume

(11) $$\frac{\pi }{3}\left|e_{ij}\right|\left(r_{i}^{2}+r_{j}^{2}+r_{i}r_{j}\right)$$

and lateral surface area (not including end caps)

(12) $$\pi \left(r_{i}+r_{j}\right)\sqrt{{\left|e_{ij}\right|}^{2}+{\left(r_{i}-r_{j}\right)}^{2}}.$$

The total volume of the cable model is estimated as the sum of all segment volumes. The total surface area of the cable model is estimated as the sum of all segment lateral surface areas, plus flat end caps for terminal segments.

We also estimate total overlap contributions to each branch point and subtract them from the totals; this estimate is computed by approximating the overlap at each branch node as a quarter-ball with radius equal to that of the vertex.


(13) $$V_{\text{overlap}}\left(r\right)=\frac{1}{3}\pi r^{3}\;A_{\text{overlap}}\left(r\right)=\pi r^{2}$$

The MASCAF Python code includes a geometric validation system that compares the surface area and volume of the mesh to those of the cable model. We also verify topological genus: for all toric spines processed in this work, CGAL-TSMS preserved the topological genus of the input surface, such that the number of independent cycles in the resulting cable graph equals the genus of the mesh. This is consistent with the topological guarantees of mean curvature flow skeletonization (Tagliasacchi et al. 2012).

As a representative example, we report validation metrics for toric spine #1 (32 cable nodes, 32 edges, fitted from a mesh with 2017 vertices and 4040 faces). Surface area matching is exact by construction: the global radius scaling step normalizes all segment lateral surface areas to match the mesh surface area, yielding a relative surface area error of 0% (without overlap correction). Without overlap correction, the total cable model volume overestimates the mesh volume by 16.1% (ratio 1.16), as expected from the systematic volumetric overlap at branch nodes. When overlap volumes are subtracted using the quarter-ball approximation described above, the corrected cable model volume overestimates the mesh volume by only 3.3% (ratio 1.03).

### 3.2 Simulation Validation

To verify the compatibility of generated cable models with multicompartmental simulation, reconstructed toric spine morphologies were imported into Arbor and simulated under standard current-clamp and alpha-synapse stimulation. The objective of these simulations was to confirm that morphologies produced by the MASCAF pipeline could be stably instantiated, discretized, and simulated without numerical or topological failure. Passive membrane parameters were assigned uniformly across compartments, and membrane potential probes were placed at every compartment to evaluate voltage propagation throughout the model.

**Figure 8:**
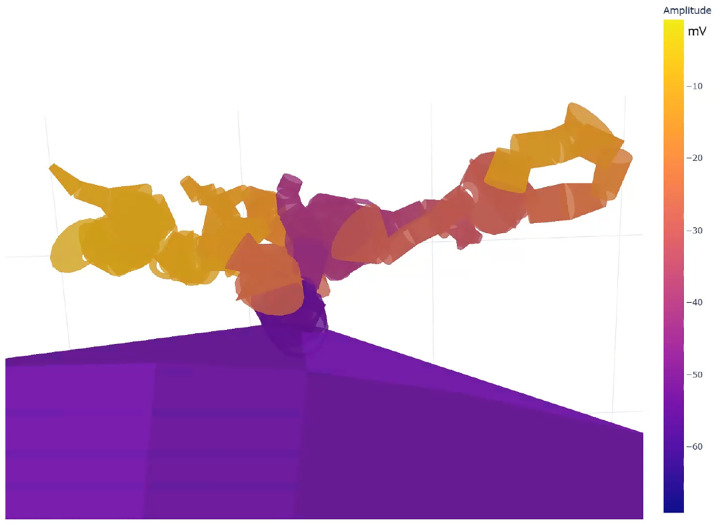
Still from an Arbor simulation of a toric spine. The color indicates membrane potential in response to synaptic inputs on the spine.

The resulting simulations exhibited stable electrotonic membrane dynamics and continuous voltage propagation across all tested morphologies, including cyclic regions of the toric spines. With neck coordinates retrieved from imaging data, we attach a large sink volume to the spine model at that point to allow for realistic electrical diffusion. A snapshot of one such simulation is shown in Figure 8. Locations of 25 active post-synaptic densities were retrieved from imaging data and recreated with alpha-synapse models.

No simulator instability, disconnected compartments, or topology-related discretization failures were observed during model initialization or time integration. Voltage responses followed the qualitative behavior expected from passive cable theory, with attenuation and temporal smoothing increasing as a function of path length and branching complexity.

**Figure 9:**
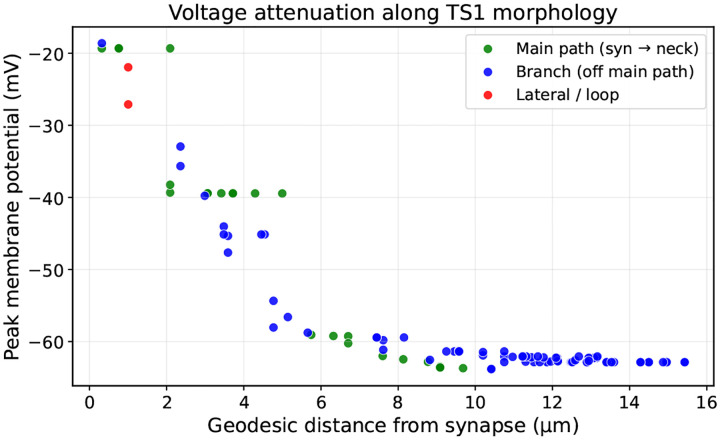
Peak membrane potential vs. geodesic distance from a distal alpha-synapse stimulus site in a passive simulation of toric spine #1 using Arbor. Monotone decay is consistent with electrotonic attenuation in a passive cable model.

To further validate the cable models quantitatively, we measured peak membrane potential at every compartment as a function of geodesic (path) distance from the stimulus site, under passive membrane parameters with a single alpha-synapse stimulus. Figure 9 shows the resulting attenuation profile for toric spine #1. Peak voltage decays monotonically with path distance, consistent with electrotonic attenuation predicted by passive cable theory and confirming that the fitted cable model geometry produces physiologically plausible electrical behavior.

These results demonstrate that cable models generated using MASCAF are compatible with modern multicompartmental simulators and produce electro-physiological behavior consistent with passive cable theory, validating both topological and geometric fidelity.

## 4 Discussion

We present the Mesh and Skeleton Cable-model Fitting (MASCAF) pipeline for fitting cable models (e.g., SWC) to 3D surface meshes, particularly suited for topologically complex neuronal structures. Cable models, like the SWC format, are the most common morphology specification used in multi-compartmental simulators like NEURON and Arbor, due to the simple application to the cable equations. Development of MASCAF was motivated by the notable discovery of a new kind of dendritic spine on auditory space-specific neurons in the barn owl’s midbrain. Dubbed toric spines (Sanculi et al. 2020), they prominently feature topological loops, the function of which are unknown. Cyclic topology of these morphologies breaks the assumption of tree-like neural structures. We showed how the CGAL Triangulated Surface Mesh Skeletonization operation is used to robustly compute midline skeletons for surface meshes via mean curvature flow, which preserves the topological genus of the surface as the number of cycles in the cable graph. The MASCAF Python code (Fox 2026) uses the combined mesh and skeleton data to fit the cable graph and write it to an SWC file, and also includes tools for quality optimization of the input skeleton and output cable graph. For cyclic topologies, the output file is a valid arborescence (a rooted, directed tree) with cycle-closure directives carried in the file header; these can be read during the simulation phase to establish electrical connections, thereby preserving the real morphology in multicompartmental simulations.

### 4.1 Parameters, Limitations, & Opportunities

The most important global parameters of the MASCAF pipeline are

- $w_{H}$ : quality_speed_tradeoff in CGAL-TSMS
- ℓ : maximum edge length in the cable fitting algorithm.

As discussed in Sections 2.2 and 2.3.1, $w_{H}$ controls both the small-scale quality of the skeleton and the branching density. For small values of $w_{H}$, the skeleton may not follow the midline well, while for large values, the skeleton may include spurious branching. This is a limitation of the pipeline because, in general, the user must run CGAL-TSMS for several values of $w_{H}$ and choose one based on visual accuracy. However, it seems likely that a robust oracle system could be developed to measure geometric properties of the mesh and suggest a value. This aspect will be addressed in future development.

The maximum edge length ℓ presents a similar issue. If the value is too small, then the dimension of the cable equations becomes large, and thus the simulation cost grows. If the value is too large, then the model and simulation may not be a realistic representation of the biological system. But, like $w_{H}$, a general heuristic or oracle system could be developed. These constitute opportunities for future research and software development.

### 4.2 Future Work

The development of MASCAF has solved a major roadblock in the detailed analysis of auditory space-specific neurons (SSNs) in the midbrain of the barn owl. High-resolution imaging data has provided unprecedented levels of detail in the anatomy and morphology of these systems, but construction of morphologically accurate simulations from that data has proven challenging. With this problem resolved, the next step involves constructing explanatory and predictive models for toric spines and SSNs. With MASCAF, we are able to perform simulations in Arbor of isolated toric spines with realistic synaptic and current mechanisms, probing the dynamics of signal integration. For the full SSN model, we take a multi-scale approach, combining cable models of individual toric spines onto cable models of whole neuron membranes. In this way we may reconstruct large and small scales at high resolution, and then probe full-scale simulations to illuminate the function of toric spines as components of a sophisticated neural computation system.

## Figures and Tables

**Figure 4:**
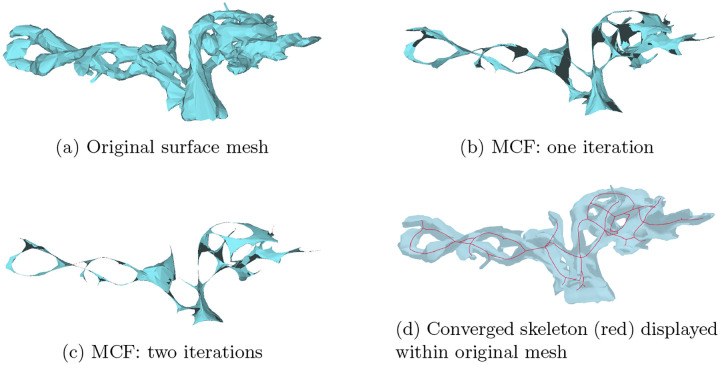
Snapshots of mean curvature flow skeletonization (MCFS) applied to a toric spine mesh with many loops.

**Figure 5:**
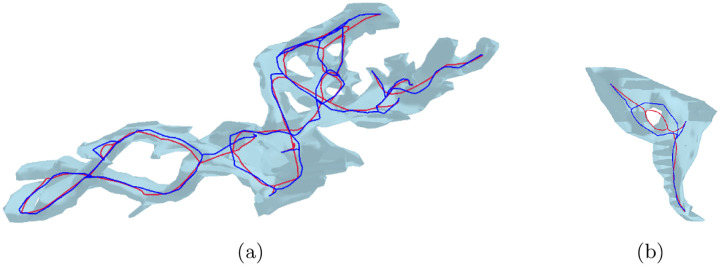
Two toric spines meshes rendered with skeletons. Skeleton vertices directly from the CGAL-TSMS calculations are shown in red, and the refined placements using MASCAF’s basis optimizer are shown in blue. Note that in (b) the original skeleton (red) is not fully contained within the mesh, while the optimized result (blue) is.

**Figure 10:**
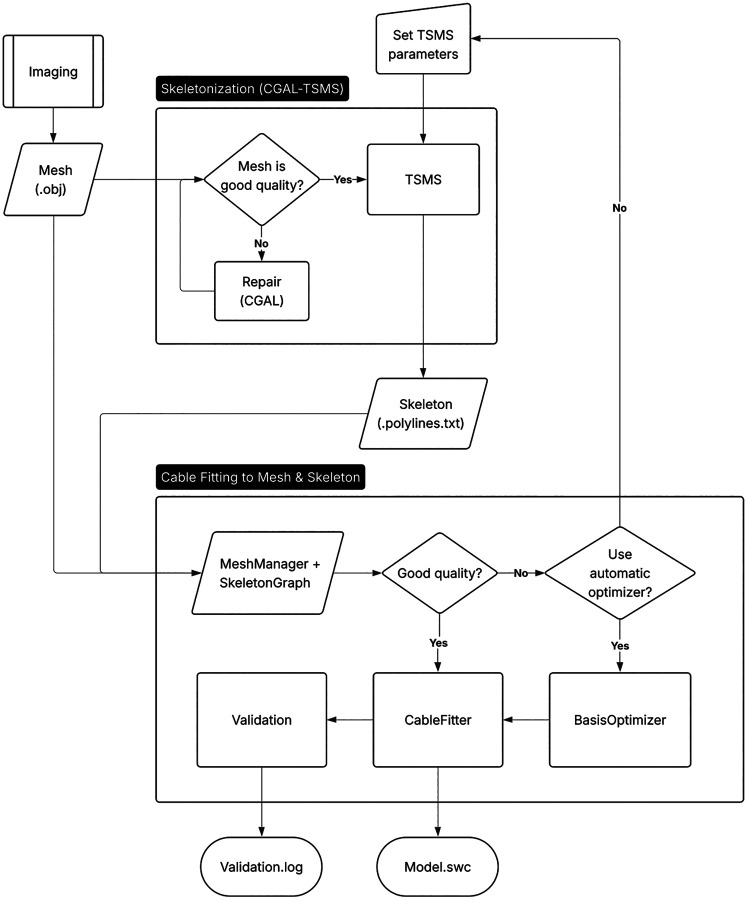
The MASCAF pipeline.

**Figure 11:**
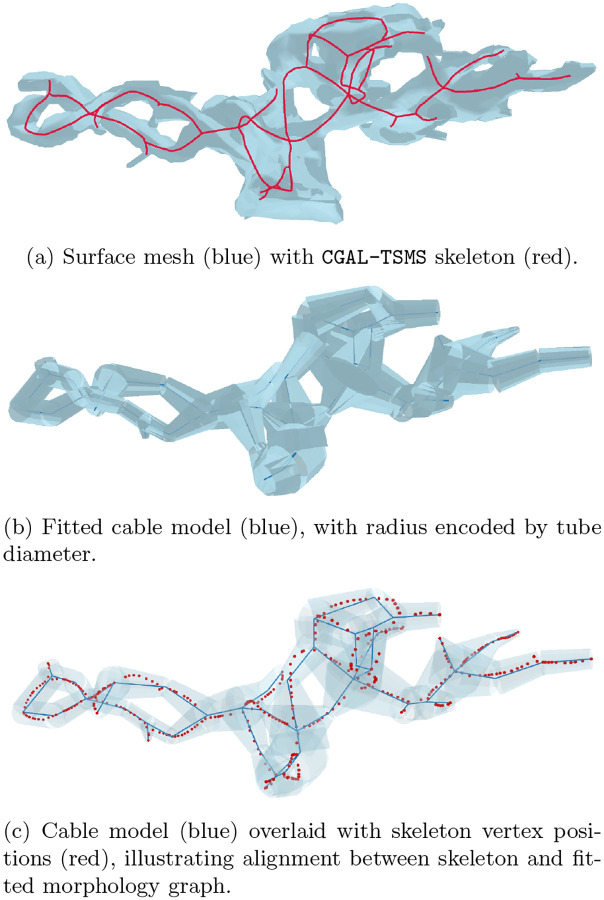
Toric spine #1. In all panels, the surface mesh is shown in blue and skeleton vertices in red. This spine has 6 loops.

**Figure 12:**
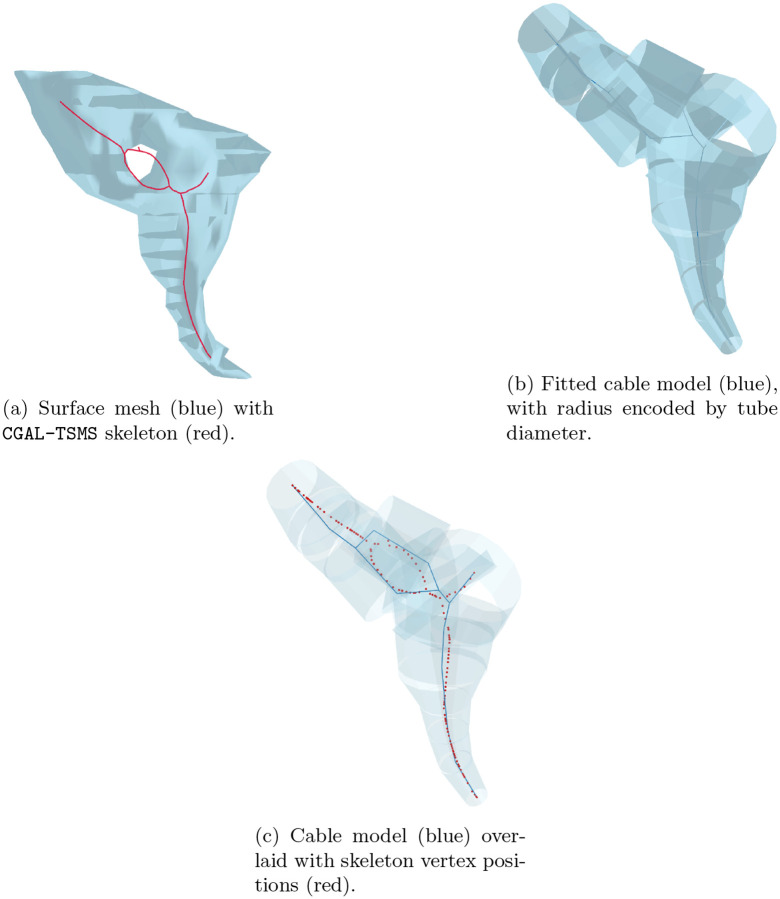
Toric spine #2. In all panels, the surface mesh is shown in blue and skeleton vertices in red.

**Figure 13:**
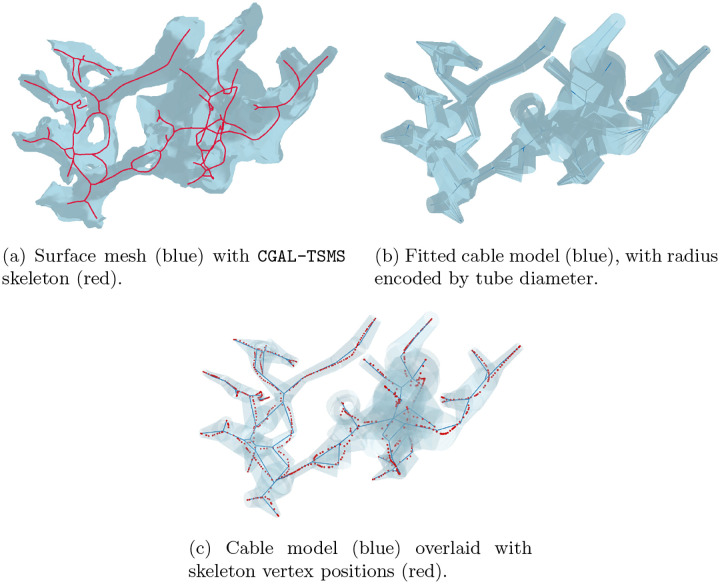
Toric spine #3. In all panels, the surface mesh is shown in blue and skeleton vertices in red. This spine has 9 loops and is the most topologically complex in the dataset; its full connectivity is not easily visible from a single viewpoint.

**Figure 14:**
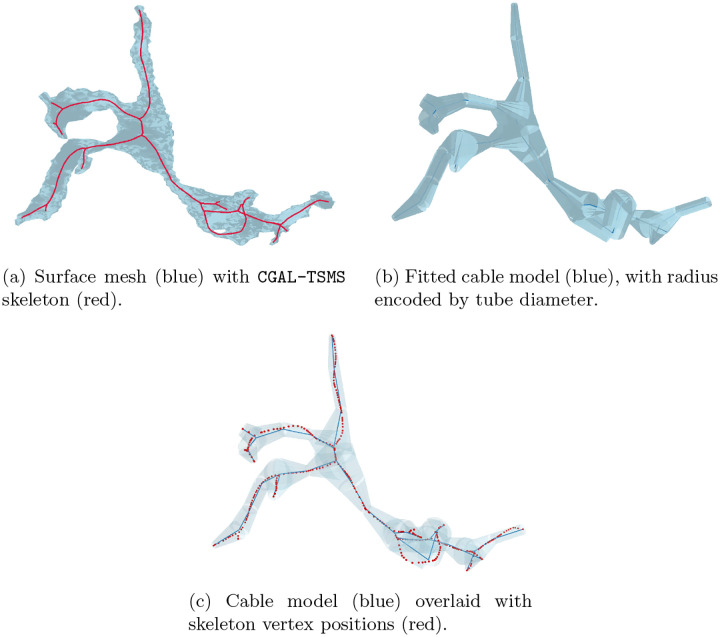
Toric spine #4. In all panels, the surface mesh is shown in blue and skeleton vertices in red.

**Figure 15:**
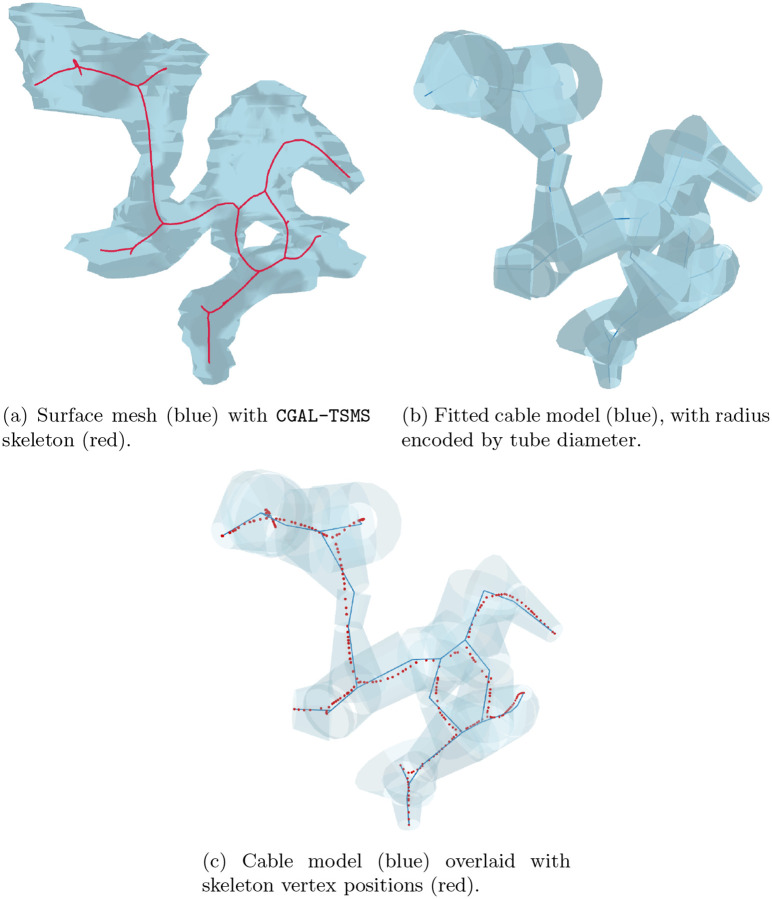
Toric spine #21. In all panels, the surface mesh is shown in blue and skeleton vertices in red.

## Data Availability

MASCAF Python code: https://github.com/jmrfox/mascaf

## B Related Software Tools

Commercial platforms such as Imaris (Oxford Instruments n.d.) provide powerful workflows for fitting cable models directly to imaging data and are widely used to reconstruct neuronal morphologies and connectomes from microscopy data. These tools are primarily designed for voxel-based image data and assume tree-like structures, limiting their applicability to exotic morphologies. Unfortunately, their proprietary nature restricts accessibility, reproducibility, and software interfacing.

Vaa3D (Peng, Bria, et al. 2014), a multi-feature open-source program for imaging data manipulation, supports some neuron reconstruction from volumetric datasets and provides cable-model outputs compatible with standard simulators. These tools are optimized for tracing neuronal arbors from image stacks and large-scale connectomics datasets. However, they largely operate on voxel representations and assume tree-like structures, making them unsuitable for direct processing of particularly complex surface meshes.

A related class of approaches is voxel-based medial-axis methods, most notably the Tree-structure Extraction Algorithm for Accurate and Robust Skeletons (TEASAR) (Paik et al. 2001) and its derivatives. TEASAR-based methods operate on labeled volumetric segmentations and iteratively extract centerlines by computing shortest paths to the surface in a grid. Modern implementations such as Kimimaro (Silversmith, Bae, et al. 2021) extend this approach to large-scale dense connectomics datasets by incorporating efficient graph traversal and anisotropic distance transforms. Kimimaro is particularly designed to skeletonize large volumetric segmentations with high throughput and robustness. These methods are closely integrated with Google’s Neuroglancer (Maitin-Shepard et al. 2021) ecosystem, including tools such as Igneous (Silversmith, Zlateski, et al. 2022) for distributed data processing and CloudVolume (Tartavull and Silversmith 2019) for scalable data access and visualization. Within this framework, skeletonization is performed directly on voxel segmentations, often using chunking and stitching strategies to handle large datasets. Despite their strengths, TEASAR-based approaches differ fundamentally from the approach presented here in both input representation and modeling assumptions. These methods operate on discrete voxel grids and produce skeletons that are inherently dependent on the resolution and discretization of the volumetric segmentation. While TEASAR-based methods can estimate local radius using distance-to-boundary fields, they do not directly fit cable models suitable for multicompartmental simulation.

The Coverage Axis approach (Wang et al. 2024), also medial-axis-based, provides an alternative strategy for extracting skeletal representations directly from surface meshes and point clouds. These methods aim to compute a centered and well-covered medial structure by combining geometric and sampling-based criteria, and have been shown to produce high-quality skeletons for complex shapes. However, such approaches typically require additional preprocessing, parameter tuning, or auxiliary representations beyond the surface mesh alone, complicating their integration into a streamlined reconstruction pipeline.

Importantly, although we focus on a single skeletonization algorithm in this paper, MASCAF can accept any polyline-based skeleton representation as input, regardless of the method used to compute it. This modular design allows alternative skeletonization methods, including other medial-axis approaches, to be incorporated into the pipeline if desired, while retaining the advantages of robust radius estimation and simulator compatibility.
